# Application of Bioelectrical Impedance Analysis in Weight Management of Children with Spina Bifida

**DOI:** 10.3390/nu16183222

**Published:** 2024-09-23

**Authors:** Joanna Bagińska-Chyży, Agata Korzeniecka-Kozerska

**Affiliations:** Department of Pediatrics and Nephrology, Medical University of Białystok, 17 Waszyngton Str., 15-274 Białystok, Poland; iklinped@umb.edu.pl

**Keywords:** weight management, spina bifida, body mass index, bioelectrical impedance analysis

## Abstract

Background: Children with spina bifida (SB) face an elevated risk of obesity, which necessitates precise methods for assessing body composition to ensure effective weight management. Conventional measures like BMI are inadequate for this population because of variations in growth patterns and skeletal structure. Bioelectrical impedance analysis (BIA) is a method that offers a clearer picture of body composition, yet its use in children with SB remains underexplored. Methods: Conducted on 57 children with SB and 28 healthy controls, with a median age of 11 years, this study evaluated anthropometrics, including BMI and BIA-derived metrics. The Hoffer’s scale to assess physical activity was applied in SB children. Results: Results showed that while 32% of SB patients were classified as overweight or obese based on BMI, 62% exhibited high body fat percentage via BIA. Fat-free mass, muscle and fat mass, and fat-to-muscle ratio (FMR) differed significantly compared to the reference group. Non-ambulators showed a higher median body fat mass percentage (25.9% vs. 17.8%, *p* = 0.01) and FMR (0.92 vs. 0.44, *p* = 0.003) in comparison to the community walkers. Conclusions: In SB children, BIA-measured fat mass is a better obesity indicator than BMI. Non-ambulatory, SB patients with obesity had the highest FMR values, indicating a higher risk for metabolic syndrome.

## 1. Introduction

Congenital spina bifida (SB) is a neural tube defect that arises when the spine and spinal cord fail to develop properly during the early stages of pregnancy. This condition can lead to varying degrees of physical and neurological impairments, affecting mobility. Beyond these motor function impairments, children with SB are prone to various health problems affecting their neurological and musculoskeletal systems [1]. Accurate evaluation of body composition parameters is crucial for monitoring children with SB, as body composition changes may further complicate their health, increasing the risk of obesity, cardiovascular diseases, and metabolic disorders [2]. 

Common anthropometric measurements, such as body mass index (BMI) or weight-for-height ratios, are frequently utilized to estimate body fat and nutritional status in pediatrics. Due to altered growth patterns and body posture in children with SB, these metrics are poor predictors of body fat and have limited utility in guiding nutritional interventions. The presence of scoliosis or joint contractures often complicates obtaining precise height measurements, thereby diminishing the accuracy of BMI. Moreover, BMI fails to distinguish between lean and fat mass, further limiting its effectiveness [3]. While all methods for assessing body composition have inherent limitations, dual-energy X-ray absorptiometry (DXA) is widely regarded as the gold standard for body composition measurement. However, DXA has its own constraints, including its lack of portability, high cost, and the low-level radiation exposure involved. In contrast, bioelectrical impedance analysis (BIA) is relatively simple, fast, and non-invasive, and unlike DXA, it involves no radiation exposure. This makes BIA particularly suitable for repeated measurements over time when tracking changes in body composition is required. It has been widely used in various clinical fields, offering high accuracy and efficiency at a relatively low cost [4,5,6]. Studies have shown that BIA is particularly useful in assessing body composition in infants and young children, providing important insights into early childhood development and growth. In pediatric populations with specific health concerns, such as obesity, BIA helps clinicians better understand fat distribution and muscle mass, offering more detailed information than BMI alone. This has led to a more refined approach in managing childhood obesity, especially in cases where traditional methods may overlook nuances like hydration status, as noted in recent findings [7,8]. BIA devices measure the resistance of body tissues to a small, safe electrical signal, allowing for the estimation of, for example, fat-free mass, body fat, and muscle mass. Despite widespread use of BIA, changes in body composition in children with SB are not well characterized. This gap in knowledge highlights the need for further research. 

This study aims to investigate body composition in pediatric patients with SB, with a focus on the limitations of traditional BMI measurements and the potential benefits of alternative method: BIA. The specific objectives are to

1. Assess the prevalence of obesity in children with SB using both BMI and BIA.
2. Explore the limitations of BMI in accurately reflecting the total body mass of SB patients.
3. Evaluate the effectiveness of BIA as a more accurate, non-invasive method for assessing body composition in children with SB.
4. Compare the body composition of pediatric patients with SB to the reference group.

## 2. Materials and Methods

### 2.1. Patients

Cross-sectional data from a prospective cohort study were analyzed. This study involved 85 participants: 57 children diagnosed with congenital SB and 28 healthy volunteers who visited a pediatrician for balance tests aged 1–18 years and were without any known chronic health conditions. We measured body composition using two indirect methods, anthropometry and BIA, correlating these measurements with BMI to evaluate the concordance between BMI and fat mass in children with SB. The lesion level of SB patients was reported intraoperatively and radiologically and scored from 1 to 3 (1—thoracolumbar, 2—lumbosacral, and 3—sacral lesion). The ambulatory function of the SB patients was classified using the Hoffer’s scale (HS) into the categories of non-ambulators, exercise walkers, household walkers, and community walkers [9]. Exercise walkers, who walked only during therapy, and household walkers, who used braces or crutches for indoors and wheelchair outdoors, were grouped with non-ambulators, who were entirely wheelchair-dependent, due to their similarly low physical activity in daily life.

### 2.2. Anthropometrics

The weight and height of each participant were recorded. For participants with SB who depended on a wheelchair, weight was measured using an electronic scale. The participant, seated in the wheelchair, was wheeled onto the scale, and the weight of the wheelchair was subtracted from the total to determine the individual’s weight. Height for these participants was measured in a lying-down position. Body surface area (BSA) was calculated with the Dubois formula: BSA = height^0.725^ × weight^0.425^ × 0.007184.

BMI was calculated using the standard formula: the body mass divided by the square of the body height, expressed in kg/m^2^. The corresponding BMI-for-age percentiles based on Centers for Disease Control and Prevention growth charts for children ages 2–19 years were obtained [10]. BMI percentile categories were as follows: underweight < 5th percentile, normal weight from 5th–85th percentiles, overweight from 85th–95th percentiles, and obesity equal to or greater than the 95th percentile. Additionally, we converted BMI to BMI standard deviation scores (Z-BMI) and classified the results according to the World Health Organization age- and gender-specific growth standards [11]. Underweight was defined as a BMI z-score < −2 standard deviations (SD), normal weight as a z-score between −2 SD and +1 SD, overweight as a z-score >  +1 SD, and obesity as a z-score > +2 SD.

### 2.3. BIA

Whole-body BIA measurements were performed using the BioScan 916S device (Maltron International, Essex, UK) according to the manufacturer’s instructions. This device delivers a current of 0.7 mA with an impedance of 100 to 1000 Ω, precision of ±3 Ω, and a frequency of 50 Khz. The measurement was applied on the right side of the body with the participant lying supine. Body composition measurements of fat-free mass (FFM, kg and %) consisted of body cell mass (kg) and extracellular solids (kg); the fat mass (kg and %), muscle mass (kg and %), total body water (TBW, lt and %) intracellular water (ICW, lt and %), extracellular water (ECW lt and %), protein mass (kg), mineral mass (kg), total body calcium (g), and total body potassium (g) were obtained from BIA data, resistance, and reactance. The BIA-derived body fat percentage was applied to body fat percentile reference curves for children aged 3 to 16 years [12]. Due to these age limitations, the reference curves did not encompass the entire study sample, resulting in the absence of fat mass percentiles for 4 children younger than 3 years of age. For participants older than 16 years of age, we used the adults’ cut-offs for high body fat percentage proposed by Macek et al. [13], established as 25.8% for men and 37.1% for women. We used 3 body fat mass categories, as follows: low < 10th percentile, normal between 10th–90th, and overweight and obese above 90th and 97th body fat percentiles, respectively. The fat-to-muscle ratio (FMR) was determined by dividing body fat mass (kg) by muscle mass (kg) [14].

### 2.4. Statistics

The data were collected in a Microsoft Excel database. Statistical analysis was performed using Statistica 13.0. (StatSoft Inc., Tulsa, OK, USA). Continuous variables were expressed as the median and range, unless stated otherwise. All studied parameters were analyzed using nonparametric tests: Mann–Whitney, Kruskal–Wallis, and Chi2 analysis. Correlations were assessed with the Spearman test. Values of *p* < 0.05 were considered significant.

### 2.5. Ethical Issues

This study was approved by the Ethics Committee of the Medical University of Bialystok, which complies with the World Medical Association Declaration of Helsinki regarding the ethical conduct of research involving human subjects and/or animals. Patients and their caregivers were enrolled into the study after obtaining informed consent.

## 3. Results

The cohort consisted of 47/85 (55%) girls and 38/85 (45%) boys, with a median age of 11 years (2–18 years). There was no statistically significant difference between SB children and healthy control groups as regards to age, gender, body weight, BMI, and BSA except height measurements. This was due to the shorter vertebral dimension and malformations of the bone structure due to SB [15]. In the SB patient group, FFM, muscle mass, percentage of fat mass, FMR, TBW, ICW, protein, and mineral mass, including total body potassium, had significant changes when compared to the control group (*p* < 0.05). Statistically significant positive correlations were recorded between mineral mass and BMI (r = 0.571), fat mass in kg (r = 0.509), muscle mass in kg (r = 0.89) and % (r = 0.62), and total body calcium (r = 0.861). Table 1 shows the anthropometric and bioimpedance characteristics of both studied groups.

### 3.1. The Agreement between BMI and Body Fat Percentage

The median BMI was similar in both groups (17.8 kg/m^2^ in SB vs. 17.9 kg/m^2^ in the reference group), but the median body fat mass percentage differed significantly (23.7% in SB vs. 19.1% in the reference group, *p* < 0.001). According to BMI measurements, 29 out of 53 (55%) of the children with SB were categorized as “unhealthy”, having either excessively high or low BMI. When evaluated by fat mass percentage, 41 out of 53 (77%) were found to have abnormal fat mass percentages, either excessively high or low. Although 18 out of 57 (32%) SB patients were classified as overweight or obese based on traditional BMI criteria, 30 out of 53 (62%) exhibited body fat percentages, measured via BIA, exceeding the 90th percentile. Table 2 illustrates the concordance between BMI categories and body fat percentage categories in both studied groups, detailing the number and percentage of children falling into each BMI and fat mass category. 

The body fat mass in SB patients exhibited significant variability, even among children with comparable BMIs, demonstrating considerable inter-individual differences. There was a significant positive correlation between BMI percentiles and fat mass in kg and % (r = 0.625, r = 0.584; respectively) in the SB group.

### 3.2. Body Composition and Daily Physical Activity in SB Children

**Figure 1:**
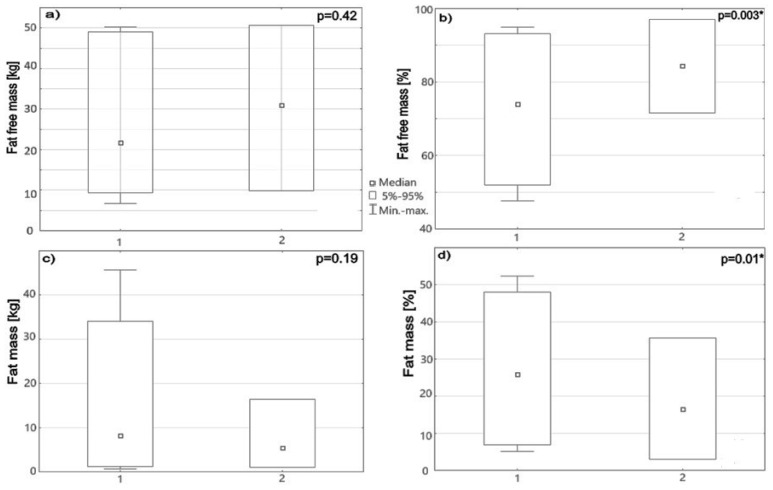
Box -whisker bars show (**a**) FFM (kg), (**b**) FFM (%), (**c**) fat mass (kg), and (**d**) fat mass (%) in SB patients divided into 2 groups: 1—low physical activity, 2—community walkers. * *p* < 0.05.

The majority of SB patients, 34/57 (60%), had lumbosacral spinal lesions, 14/57 (25%) thoracolumbar, and 9/57 (15%) sacral lesions. Among all the study subjects, 28/57 (49%) were classified as non-ambulators, 5/57 (9%) as exercise walkers, 11/57 (19%) as household walkers, and 13/57 (23%) as community walkers. When comparing anthropometric parameters between non-ambulatory and ambulatory SB children, no differences were found concerning age, heigh, weight, and BMI (*p* = 0.96, *p* = 0.49, *p* = 0.89, *p* = 0.21 respectively, *p* < 0.05), but they significantly differed with BIA parameters. Non-ambulators showed a higher median body fat mass percentage in body composition in comparison to the community walkers (25.9% vs. 17.8%, *p* = 0.01). Consequently, they had statistically significantly higher median values of FMR (0.92 vs. 0.44, *p* = 0.003). Regarding constituents of FFM, differences were observed in the percentage of muscle mass and TBW. As expected, non-ambulators had a lower median muscle mass (8.6 kg vs. 12.4 kg, *p* = 0.57) and percentage (28.2% vs. 32%, *p* < 0.001), as well as lower TBW in lt and % (17.4 lt vs. 22.7 lt, *p* = 0.64; 56% vs. 60%, *p* = 0.03), than the community walkers. More details are presented in Figure 1.

## 4. Discussion

The global rise in childhood obesity has been described as a pandemic and is considered one of the most pressing health issues of the twenty-first century. Focusing on children and adolescents is crucial, because those who become overweight or obese early in life are about five times more likely to remain so into adulthood [16]. Children with disabilities, including SB, are in a high-risk group, being two to three times more likely to become overweight or obese than their typically developing peers [17]. Hence, effective weight management is essential for providing high-quality health care to SB children.

Several studies have attempted to determine the obesity rates among children with SB, finding that its prevalence ranges from 18% to 50% [18,19,20]. A major concern is that these studies define obesity in children based on a certain BMI percentile for age and gender. However, using BMI in this patient population is controversial and there is currently no accepted standard to identify obesity [21]. First, height measurements in children with SB differ significantly from those in non-disabled children, which makes the use of BMI problematic. Factors such as lower limb and trunk hypoplasia, vertebral anomalies, and paralytic musculoskeletal deformities cause short stature, which was also observed in our study sample. Secondly, BMI does not directly measure body fat and its regional body distribution [3,22]. In our study, both cohorts exhibited median BMI percentiles within the normal range. However, traditional BMI criteria classified one-third of SB patients as overweight or obese. This proportion nearly doubled when body fat mass, assessed through BIA, was applied. Such discrepancies were not present in the reference group, where a strong concordance between BMI and fat mass categories was observed. The study by Liu J.S. et al. [23] expands the debate on the problems related to BMI as a marker of excess adiposity in SB. The investigation was conducted in small cohort of SB patients, including 18 adults, who underwent obesity classification using BMI by length and arm span, abdominal girth, and percent trunk fat on DXA. Using BMI categories, 43.8% of subjects were classified as obese. However, alternative measures, such as anthropometric assessments focusing on body composition and fat distribution, revealed a higher obesity rate. Trunk fat percentage by DXA categorized 83.3% of subjects as obese. In our study, we utilized different method to assess body composition, BIA, yet the conclusion remains unchanged. Employing methods that more accurately evaluate true body composition in the SB population raises the number of individuals classified as obese. This suggests that a significant portion of subjects deemed to have a normal BMI by conventional standards may, in fact, be obese.

The life expectancy of individuals with SB has significantly improved, rendering it a life-long condition. From a clinical perspective, it is crucial for healthcare providers to monitor and manage long-term complications. Obesity has been identified as a primary factor contributing to the onset of metabolic syndrome. In a study analyzing 34 adolescents with SB, the prevalence of metabolic syndrome was found to be 32.4%, with a higher incidence observed among obese individuals [24]. In this paper, we applied the FMR, an alternative approach for assessing metabolic syndrome, which has been validated in adults. The higher the FMR levels, the greater risk of developing conditions like type 2 diabetes, cardiovascular diseases, and increased mortality. The recent study by Zhang JX et al. [25] showed a positive correlation between dyslipidemia and high FMR values in transitional-age youth. Our findings indicated that children with SB had higher FMRs than the reference group. Those who were non-ambulatory and obese had the highest FMR values. This suggests that these specific subgroups of SB patients are at a heightened risk for metabolic syndrome and its long-term health implications. While little is currently known about FMRs in children, our study aims to explore its potential use, though additional studies are needed to confirm our findings.

Both obesity and osteoporosis are complications associated with SB, putting these patients at risk of bone disease [26]. Our study found that children with SB had significantly lower mineral mass compared to healthy controls. Interestingly, we observed a positive correlation between mineral mass and BMI, fat mass, and muscle mass, suggesting that obesity might have a protective effect on mineral mass. This finding aligns with a study by Trinh A. et al. [27], which reported a positive correlation between fat mass and bone mineral density obtained by DXA in adult SB patients. They concluded that higher bone mineral density in those with a higher BMI could be due to increased lean tissue or fat mass. The main takeaway is that adults with SB are at high risk for both low bone density and obesity, along with related comorbidities. Our findings suggest that this process may begin as early as childhood.

The present study has several limitations. First, the sample size of 57 children with SB may be considered relatively small. However, it is important to acknowledge that SB is a rare congenital condition, and there is a global trend toward pregnancy termination following prenatal diagnosis, further reducing the available population for study. Multi-center collaborations would help mitigate that issue, enabling researchers to pool data from different regions. Second, as the data were obtained from a single, large tertiary referral center, the findings may not be fully generalizable and require external validation. Additionally, body composition was assessed using two indirect methods: anthropometry and BIA. While these methods are widely used, more direct methods, such as DXA and magnetic resonance imaging (MRI), exist. DXA is regarded as the gold standard for detailed body composition assessment; however, its high cost, limited availability, and exposure to radiation limit its practicality. MRI provides detailed imaging of soft tissues, including fat and muscle, but it also has limitations, especially in pediatric populations. MRI is expensive, time-consuming, and often restricted to specialized clinical or research environments, making it less feasible for use in large-scale studies. Moreover, younger children may struggle to remain still during the scan, leading to motion artifacts that compromise the accuracy of the measurements.

Future research could incorporate direct techniques to offer a more comprehensive understanding of body composition in children with SB. Emerging imaging technologies or novel non-invasive methods that address the drawbacks of DXA and MRI could also be explored. Despite being less precise, BIA is more practical for regular monitoring due to its simplicity and non-invasiveness, though its accuracy can be affected by factors such as hydration status and body composition. A key limitation of BIA is its tendency to overestimate body fat in obese individuals due to the reliance on electrical conductivity, as fat tissue has higher resistance to electrical currents. This can result in inflated body fat estimates in individuals with greater fat mass, though it tends to provide more reliable estimates in lean populations, such as the children studied in our research. BIA was selected for this study based on its practicality. Regular monitoring of body composition in conjunction with functional and metabolic outcomes may offer deeper insights into the unique growth patterns in children with SB, aiding in personalized clinical care and interventions.

## 5. Conclusions

In conclusion, this study emphasizes several key findings related to obesity and body composition in children with SB:

(i) Childhood obesity is a significant global concern, and children with SB are at an even higher risk due to altered body composition and physical limitations.
(ii) Traditional BMI measurements may underestimate obesity in SB patients, as they do not account for the unique body composition seen in this population, particularly the imbalance between muscle mass and fat tissue.
(iii) BIA offers a non-invasive and more accurate method for assessing body composition in SB patients, revealing higher obesity rates compared to BMI alone.
(iv) Obesity in SB patients is associated with an increased risk of metabolic syndrome and bone disease, emphasizing the need for early intervention and the use of precise assessment methods to manage these risks effectively.

## Figures and Tables

**Table 1:** Anthropometric and bioimpedance traits for participants.

Variables	SB Patients	Reference Group	*p* Value
	*n* = 57	*n* = 28	
**Anthropometric parameters (median, min.–max.)**			
Height (cm)	132 (70–174)	154 (108–181)	0.02 *
Weight (kg)	33 (8.5–95)	42.6 (17.5–77.5)	0.19
BMI (kg/m^2^)	17.8 (10.4–35.3)	17.9 (13.1–23.6)	0.68
BMI (centiles)	52.6 (0–100)	52.7 (1.6–76.7)	0.79
Z-BMI	0.06 (-8–3.5)	0.06 (-2.1–0.7)	0.85
BSA (m^2^)	1.1 (0.3–2)	1.36 (0.72–2)	0.1
**BIA and body composition (median, min.–max.)**			
FFM (kg)	22.3 (6.7–50.7)	35.6 (14.2–64.8)	0.03 *
(a) body cell mass (kg)	11.4 (2.6–32.5)	18.4 (6.5–33.7)	0.04 *
(b) extracellular solids (kg)	10.3 (4.1–25.9)	15.7 (7.5–31.1)	<0.001 *
FFM (%)	76.7 (47.6–96.9)	80.9 (69–95.9)	0.01 *
Fat mass (kg)	8 (0.69–45.6)	5.9 (1.4–17.6)	0.65
Fat mass (%)	23.7 (3–52.4)	19.1 (4–30.9)	<0.001 *
Fat mass (percentiles)	50 (3–97)	25 (3–75)	<0.001 *
Muscle mass (kg)	8.8 (1.9–25.4)	15.1 (4.8–32.3)	0.04 *
Muscle mass (%)	29.2 (21.2–42)	32.5 (27–41.7)	<0.001 *
FMR	0.81 (0.1–2.4)	0.55 (0.1–1.03)	0.002 *
TBW (lt)	17.9 (5.3–42.6)	25.6 (10.9–52.2)	0.04 *
(a) ICW (lt)	9.4 (2.1–28.9)	15.1 (5.9–27.3)	0.02 *
(b) ECW (lt)	7.4 (3.2–18.4)	10.2 (4.7–24.8)	0.12
TBW (%)	57.3 (39.1–79.7)	59.7 (48–82)	0.22
(a) ICW (%)	55.7 (21.3–69.5)	57.9 (52.4–66.4)	0.07
(b) ECW (%)	44.5 (30.5–78.7)	42.1 (33.6–47.6)	0.06
Protein mass (kg)	3.2 (0.91–13.5)	6.2 (2.3–16.6)	<0.001 *
Mineral mass (kg)	1.2 (0.31–4.7)	2.4 (0.9–5.8)	<0.001 *
Total body calcium (g)	363 (107–1116)	598 (231–1220)	0.06
Total body potassium (g)	54.3 (12.5–154.4)	85 (30.6–160.7)	0.04 *

SB, spina bifida; BMI, body mass index; Z-BMI, body mass index z-score; BSA, body surface area; FFM, fat-free mass; FMR, fat-to-muscle ratio, TBW, total body water; ICW, intracellular body water; ECW, extracellular body water; * *p* < 0.05.

**Table 2:** Agreement between BMI and fat mass categories in both studied groups.

	Body Fat Mass Category			
*n* (%)	Low <10 pc	Normal 10–90 pc	High >90 pc	Total
		SB patients		
**BMI category** underweight < 5 pc	3 (6)	5 (9)	3 (6)	11 (21)
normal weight 5–85 pc	7 (13)	5 (9)	12 (23)	24 (45)
overweight and obese > 85 pc	1 (2)	2(4)	15 (28)	18 (34)
Total	11 (21)	12 (23)	30 (56)	53 (100)
		Reference group		
**BMI category** underweight < 5 pc	0	2 (7)	0	2 (7)
normal weight 5–85 pc	8 (29)	18 (64)	0	26 (93)
overweight and obese > 85 pc	0	0	0	0
Total	8 (29)	20 (71)	0	28 (100)

## Data Availability

The data presented in this study are available on request from the corresponding author. The data are not publicly available for ethical and privacy reasons.
